# Artificial Intelligence in Predictive Healthcare: A Systematic Review

**DOI:** 10.3390/jcm14196752

**Published:** 2025-09-24

**Authors:** Abeer Al-Nafjan, Amaal Aljuhani, Arwa Alshebel, Asma Alharbi, Atheer Alshehri

**Affiliations:** Computer Science Department, College of Computer and Information Sciences, Imam Mohammad Ibn Saud Islamic University (IMSIU), Riyadh 11432, Saudi Arabia

**Keywords:** predictive healthcare, machine learning, deep learning, ICU, sepsis prediction, ensemble methods, federated fearning

## Abstract

**Background/Objectives:** Today, Artificial intelligence (AI) and machine learning (ML) significantly enhance predictive analytics in the healthcare landscape, enabling timely and accurate predictions that lead to proactive interventions, personalized treatment plans, and ultimately improved patient care. As healthcare systems increasingly adopt data-driven approaches, the integration of AI and data analysis has garnered substantial interest, as reflected in the growing number of publications highlighting innovative applications of AI in clinical settings. This review synthesizes recent evidence on application areas, commonly used models, metrics, and challenges. **Methods:** We conducted a systematic literature review between using Web of Science and Google Scholar databases from 2021–2025 covering a diverse range of AI and ML techniques applied to disease prediction. **Results:** Twenty-two studies met criteria. The most frequently used machine learning approaches were tree-based ensemble models (e.g., Random Forest, XGBoost, LightGBM) for structured clinical data, and deep learning architectures (e.g., CNN, LSTM) for imaging and time-series tasks. Evaluation most commonly relied on AUROC, F1-score, accuracy, and sensitivity. key challenges remain regarding data privacy, integration with clinical workflows, model interpretability, and the necessity for high-quality representative datasets. **Conclusions:** Future research should focus on developing interpretable models that clinicians can understand and trust, implementing robust privacy-preserving techniques to safeguard patient data, and establishing standardized evaluation frameworks to effectively assess model performance.

## 1. Introduction

Artificial intelligence (AI) and machine learning (ML) are revolutionizing predictive analytics in healthcare, offering the potential for swift and accurate predictions that can lead to timely interventions and improved patient outcomes. The integration of AI technologies is increasingly enabled by the availability of vast amounts of data from electronic health records (EHRs), wearable sensors, and advanced medical imaging. These resources empower ML models to uncover complex patterns, facilitating early diagnoses and enhancing patient management [1,2,3].

For instance, ML models are extensively utilized in critical applications such as early sepsis detection and mortality prediction within intensive care units (ICUs) [4,5,6,7,8,9,10]. They also play a vital role in forecasting heart failure and cardiovascular events in cardiology [1,11], cancer risk stratification in oncology [12], triage support in emergency departments (EDs) [13,14], and predicting diabetes and hypertension in chronic disease management [2,3]. These applications highlight the flexibility of ML in healthcare. However, they also raise concerns about domain-specific challenges and the limited generalizability of models across different clinical settings [15,16].

Despite the advancements, there remains a lack of consolidated understanding regarding the most effective ML methods applicable across different healthcare domains. Challenges such as data heterogeneity and model interpretability persist, complicating the deployment of these technologies. The efficacy of ML models often hinges on the nature of the healthcare data; for example, ensemble methods like Random Forest and XGBoost excel with structured tabular data such as EHRs, while neural networks—especially deep learning models—are more adept at handling unstructured data types, including medical images and time-series signals (e.g., heart rate or electroencephalogram readings) [17,18].

As interest in this research field continues to grow, a significant number of publications and tools have emerged, with many researchers and practitioners actively contributing to advancements in healthcare analytics. The aim of this systematic review is to synthesize findings from recent studies, categorizing the various ML applications in predictive healthcare. We aim to explore the specific areas of healthcare that are most actively using predictive models. In addition, this review will explore the most frequently employed ML algorithms in these healthcare settings. Moreover, we will discuss the challenges and limitations currently faced in the deployment of ML in healthcare. Issues related to data heterogeneity, model interpretability, and the generalizability of findings across diverse patient populations are critical barriers that must be considered. Finally, we will explore future directions for research in this field, identifying potential pathways for overcoming existing obstacles and enhancing the integration of ML into clinical workflows.

The remainder of this review is organized as follows: Section 2 presents an outline of the research methodology; Section 3 presents the results and findings of our SLR; Section 4 discusses challenges and trends in this research area while providing insights for future research; and finally, Section 5 presents the conclusion.

## 2. Methodology

This SLR aims to investigate the landscape of ML applications in predictive healthcare analytics by addressing several key research questions: RQ1 identifies the areas of healthcare care that utilize these predictive models, RQ2 explores the most commonly used machine learning algorithms, RQ3 examines the evaluation metrics typically used for performance assessments, RQ4 highlights the limitations and challenges reported in the literature, and RQ5 discusses future directions and emerging trends in the field. This Systematic review has not been registered.

Guided by these research questions, this SLR comprehensively overviews the current state of ML in predictive healthcare analytics. Specifically, it identifies and classifies the widely used ML algorithms, maps their applications across different healthcare domains, and evaluates their performance metrics. The SLR analyzes the limitations and challenges reported in the literature and outlines the emerging trends and future research directions that can enhance the development and integration of ML in healthcare systems.

### 2.1. Selection Criteria and Search Strategy

This review is based on studies published between 2021 and 2025, collected from peer-reviewed journals and conference proceedings. All eligible studies utilized predictive machine learning (ML) models in one of the following healthcare domains: cardiology, intensive care units (ICUs), sepsis, general health, oncology, COVID-19, diabetes, and emergency departments (EDs).

Studies published in languages other than English were excluded to maintain consistency in data interpretation. Additionally, we excluded ML studies that fell outside the specified healthcare domains, studies focused solely on descriptive analytics, non-peer-reviewed articles (such as blogs and white papers), as well as duplicate or non-original works.

The literature search was conducted across two major academic databases: Web of Science and Google Scholar. The search terms were carefully selected to encapsulate the intersection of machine learning and predictive healthcare. The specific search terms used were: (“machine learning” OR “ML”) AND (“predictive analytics” OR “prediction”) AND (“healthcare” OR “medical” OR “sepsis” OR “cardiology”).

For each database, the search was conducted from the start of 2021 to the end of 2025. The initial number of studies identified was 27, which were then screened for eligibility. After applying the inclusion and exclusion criteria, 22 studies were deemed appropriate for inclusion in this SLR.

### 2.2. Study Review Process and Data Extraction

**Figure 1:**
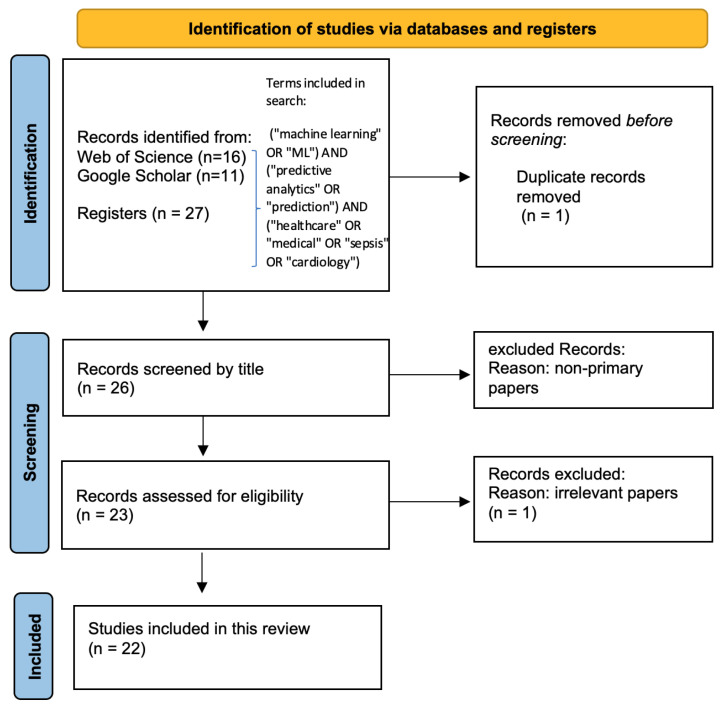
PRISMA diagram of our search process.

Three rigorous rounds of the review process ensured a comprehensive and unbiased selection of relevant articles. The entire study selection workflow is represented using the preferred reporting items for systematic reviews and meta-analyses (PRISMA) flow diagram Figure 1.

The first round involved an initial search across multiple academic databases screened through the predefined keywords, followed by automated and manual deduplication to remove redundant entries. This step ensured that each study was considered only once, preventing redundancy and maintaining the integrity of the review.

During the second round, the titles and abstracts of the remaining papers were manually screened by the research team. Non-peer-reviewed articles, non-primary research items (such as editorials and reviews), studies that did not employ ML approaches, and studies outside our targeted healthcare domains were excluded at this stage. This careful screening isolated the directly relevant and methodologically robust studies.

The third round involved a thorough full-text review of all shortlisted articles, confirming their eligibility and assessing the quality and relevance of their methodologies and findings.

The data were systematically extracted and reported in a structured Excel spreadsheet. For each selected study, we documented the year of publication, healthcare domain, study type (e.g., primary research or review), ML models and algorithms used, types of features extracted, details of the ML pipeline, datasets employed, evaluation metrics reported, main findings and contributions, reported limitations, and suggested future research directions.

To ensure traceability and facilitate future reference, we also cataloged all selected articles in a library management tool (Zotero). Importantly, each member of the review team independently extracted the data to maximize accuracy and minimize bias; any discrepancies were then discussed and collectively resolved. After this process, 22 articles were included in the final review.

Any article passing the final synthesis stage was subjected to a quality assessment based on predefined criteria. This assessment evaluated the relevance of each study to the research questions, the clarity of methodology and model explanation, completeness of the evaluation (metrics, datasets, and comparisons), publication credibility (favoring peer-reviewed journals or reputable pre-prints), and transparency of findings and reported limitations. This final screening ensured that only high-quality and reliable studies were incorporated into our final review.

## 3. Results

**Figure 2:**
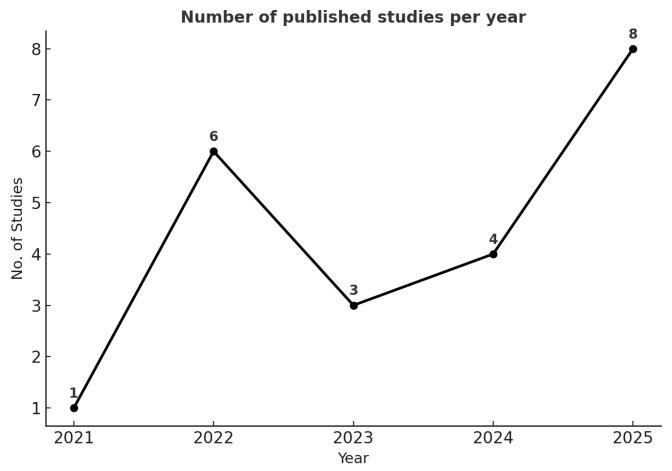
Number of studies by year of publication.

This SLR summarizes the 22 studies selected through the PRISMA process. These studies were published between 2021 and 2025, revealing clear patterns in the adoption, performance, and limitations of machine learning (ML) for predictive healthcare. Rather than isolated case studies, the evidence collectively demonstrates both convergence toward certain methodologies and divergence driven by domain-specific requirements. The graph in Figure 2 shows the number of articles that use ML and predictive analytics in healthcare between 2021 and 2025.

A single study was published in 2021, indicating that few such studies were published before 2022. The publication volume slightly increased between 2022 and 2024 and sharply increased in 2025, reflecting a rapid rise in research and interest in AI-driven healthcare prediction. The driving forces of this growth are the COVID pandemic (perhaps among the most important drivers in healthcare systems), the increasing availability of large-scale EHRs, edge computing, and real-time patient data, which have improved the robustness of model development and testing. Technological advances such as AutoML frameworks (e.g., AutoPrognosis 2.0) confirm the growing impact of ML not only in academic studies but also in real-world clinical use.

### 3.1. Healthcare Domains

The dominance of ICU and critical care studies underscores how ML is most effective where large, structured datasets (e.g., EHR, vital signs) are available and where clinical urgency demands accurate prediction. Ensemble-based models (e.g., RF, XGBoost) consistently achieved strong discriminative performance (AUROC > 0.9), yet their limited external generalizability across hospitals illustrates that data richness alone cannot ensure portability. In contrast, oncology and cardiovascular studies, though fewer in number, highlight the increasing reliance on deep learning applied to imaging and genomic data, suggesting that the complexity of input data dictates methodological choice more than domain size. Chronic disease management (e.g., diabetes) leaned heavily on IoT–ML hybrids, reflecting the necessity of longitudinal, real-world monitoring rather than acute event detection. COVID-19 models, while innovative, revealed a lack of prospective validation, raising concerns about robustness in fast-changing health crises. The 22 analyzed studies are grouped into their different domains in Table 1.

ICU and critical care emerged as the most frequently studied domains, not merely because of their clinical importance but also due to the abundance of structured, high-frequency data available in these settings. For instance, study [6] inputted real-time EHR data to ensemble-based models for sepsis-onset prediction. These applications are critical in time-sensitive environments, where early intervention significantly improves patient outcomes. However, low generalization of ML methods across institutions remains a key limitation in this domain. In the emergency department (ED), ML methods play a critical role in enhancing triage and early risk assessment, reflecting the urgent need for rapid decision-making in high-pressure environments. Study [5] leveraged the vital signs and laboratory results for deep-learning prediction of sepsis at triage. Although ML in this domain highlights the value of rapid, real-time analytics in high-pressure decision environments, it has not resolved deployment challenges such as data latency and workflow integration.

In the cardiovascular domain, ML methods have been increasingly applied to predict cardiac events, heart failure, and risk scores, reflecting the pressing need for early intervention in chronic, high-mortality conditions. For example, study [11] combined structured patient records with echocardiographic features to support early cardiovascular diagnosis. These models offer considerable promise, especially in chronic disease management, but require high-quality features and continuously monitored data.

In oncology, ML applications targeting survival estimation and recurrence prediction reflect the urgent clinical need to personalize treatment pathways and anticipate long-term outcomes. Study [21] integrated genomic data with clinical history to predict breast cancer recurrence. Although ML models have achieved high predictive performance, they are difficult to interpret and cannot adequately account for the variability of cancer subtypes.

In diabetes and other chronic diseases, the widespread adoption of ML for predicting disease onset and complications reflects the urgent need for proactive management in conditions that progress silently over time. Study [2] proposed a smart healthcare framework that detects type 2 diabetes risk based on combined clinical and behavioral data. These models are valuable for population-level screening but are less suitable for variable behavioral data and long-term monitoring.

During the COVID-19 pandemic, ML models for hospital admission prediction and severity assessment reflected the urgent demand for scalable decision-support tools under crisis conditions. Study [17] targeted pediatric COVID-19 admissions using structured EHR inputs. These models can adapt to crisis settings but often lack prospective validation.

In general healthcare and primary care, ML applications for risk scoring and decision support illustrate a shift toward preventive medicine, where early identification of at-risk patients can reduce long-term healthcare burdens. Embedding such tools in outpatient settings highlights their potential to democratize predictive analytics beyond specialized hospitals. Study [18] embedded predictive tools into primary care workflows to flag early deterioration. Although such tools enhance preventive care, they are not easily integrated into legacy health systems.

For other specific conditions such as acute kidney injury and hospital length of stay, ML applications demonstrate the versatility of predictive models in addressing diverse clinical challenges. For instance, study [7] estimated the discharge time of trauma patients using Gradient Boosting. These applications showcase the versatility of ML but require domain-specific data and close clinical alignment.

### 3.2. Applied Datasets

A sharp dichotomy emerged between reliance on public benchmark datasets (e.g., MIMIC, PhysioNet) and institution-specific data. Benchmarks promoted comparability but risked overfitting research to narrowly defined populations. Conversely, local datasets increased clinical relevance but at the expense of generalizability. This trade-off suggests that progress in predictive healthcare requires hybrid validation strategies—benchmarks for reproducibility, and multi-center datasets for population diversity. Table 2 lists the benchmark datasets and the studies in which they are referenced. Training on benchmark public datasets improves the reproducibility and across-study comparability of ML methods. In contrast, training on regional or hospital-specific datasets sacrifices generalizability to improve the local relevance.

### 3.3. Feature Extraction Methods

Feature extraction is a crucial step in the ML pipeline, particularly in healthcare where raw data must be transformed into meaningful inputs. The feature extraction methods employed in the reviewed studies are listed in Table 3 and briefly described below.

Manual statistical aggregation (e.g., mean, standard deviation, minimum, and maximum) converts time-series data such as vitals and lab results into fixed-length numerical summaries, simplifying the input data for traditional ML models [4,8,19]. Recursive Feature Elimination (RFE) iteratively removes the features that contribute little to model performance. RFE reduces overfitting and improves the efficiency of models, especially of tree-based classifiers [2,11,13].

The Least Absolute Shrinkage and Selection Operator (LASSO) shrinks the coefficients of less predictive features to zero, simultaneously performing feature selection and regularization. LASSO effectively handles high-dimensional datasets [10,16]. The Boruta Algorithm is a wrapper method built on RF, which aims to identify all relevant features rather than a minimal subset. This algorithm enhances the interpretability of the model and preserves its clinical value [7,16]. Principal Component Analysis (PCA) is a dimensionality reduction method that transforms features into new, uncorrelated components. PCA effectively compresses images and physiological data [11]. Autoencoders are deep learning models that learn the latent (hidden) features in complex inputs such as ECG signals or medical images, enabling anomaly detection and unsupervised learning [11,17].

SHapley Additive exPlanations (SHAP) interprets model predictions by quantifying the contribution of each feature. SHAP provides clinical transparency, which is especially important for black-box models such as deep neural networks [5,10,22]. Filter methods (e.g., Information Gain, Gini Index) evaluate features independently of the ML model employed. Fast and scalable, filter methods are suitable for early-stage dimensionality reduction [8].

Expert/clinically selected features are selected for their medical relevance or adherence to clinical guidelines (e.g., sequential organ failure assessment score, comorbidities), ensuring that medically validated indicators are included [4,12,14]. Feature-Aligned Transfer Learning (FATL) harmonizes the features across multiple datasets, allowing robust model training and generalization even when the feature definitions differ among [13].

As shown in Table 3, the reviewed studies demonstrate a methodological evolution: statistical aggregation and filter methods remain common in structured EHR contexts, while autoencoders, SHAP, and PCA increasingly appear in imaging, genomic, and time-series tasks. Importantly, the use of SHAP signals a growing awareness that interpretability is not optional but central to clinical trust. The heterogeneity of feature extraction highlights a persistent tension: methods that maximize accuracy often reduce transparency, whereas interpretable methods may limit performance.

### 3.4. Machine Learning Models

Seventeen ML models were identified across the reviewed studies. The models are listed in Table 4 and summarized below.

Random Forest (RF) is a widely used supervised learning method that constructs multiple decision trees during the training phase and combines their output to improve the overall predictive accuracy. In an analysis of patient outcomes in ICUs, RF classifier achieved strong predictive capabilities and reliability with a ROC AUC of 0.94 [4,8].

Logistic regression is a statistical model that estimates the probability of a binary outcome, such as yes or no. In a forecasting study of healthcare service demand based on microdata from the Turkish Statistical Institute, logistic regression yielded the highest AUC score (0.59) among several ML approaches, demonstrating the effectiveness of logistic regression in this context [8,23].

Support vector machine (SVM) is another supervised learning algorithm that seeks the optimal hyperplane between two classes by maximizing the margin between them. SVM relies on a training-data subset called support vectors. SVM has successfully identified individuals at high risk of depression from secondhand smoke in Korea based on their demographic and health features [8,23].

Extreme Gradient Boosting (XGBoost) is a powerful supervised learning algorithm built on the gradient-boosting framework. XGBoost is recognized for its efficiency and high performance on large datasets. XGBoost ranked among the most accurate predictive models in the above-mentioned Turkish healthcare study [23].

Neural networks are prominent supervised deep-learning models, especially suitable for high-dimensional and complex datasets. Neural networks have effectively detected subtle cardiac anomalies in medical imaging data [8,11].

Decision trees are simple yet effective supervised models that recursively split the data based on feature values. Their structure resembles a tree with internal nodes representing decision rules. Based on demographic variables, decision trees have accurately predicted the risk of acute respiratory infections among Ethiopian children under five [4].

Gradient Boosting, a boosting technique used in both classification and regression tasks, builds models sequentially to minimize prediction errors. This approach was adopted in a Sri Lankan study aimed at improving mental health predictions among refugee populations. Gradient Boosting achieved higher accuracy in a sensitive health domain [23].

Naive Bayes classifiers are probabilistic supervised algorithms that perform well when the features are conditionally independent. Both Bernoulli Naive Bayes and Gradient Boosting achieved notable performance in a study on clinical outcomes (mortality and hospital stay), proving particularly successful in mortality prediction [19].

k-Nearest Neighbors (k-NN) is a simple yet effective supervised algorithm that assigns a label to a data point based on the majority class among its nearest neighbors. k-NN has successfully detected and classified diabetes in a study based on Indian demographic and health data [8,23].

Multi-Layer Perceptron (MLP) is a feedforward artificial neural network comprising input, hidden, and output layers. MLP has effectively balanced the sensitivity and accuracy performances in sepsis-outcome predictions based on high-dimensional clinical datasets [8].

Light Gradient Boosting Machine (LightGBM) is an optimized gradient-boosting algorithm that improves the accuracy and training speed of Gradient Boosting through a leaf-wise tree growth approach. LightGBM has analyzed the age, history, lab results, and other data of patients for disease risk predictions [4].

Convolutional Neural Networks (CNNs) are a class of deep learning models designed for spatial data processing. These models have effectively handled medical imaging tasks such as echocardiographic (ECG) image analysis for cardiac assessment [11]. Recurrent Neural Networks (RNNs), which process sequential data and capture temporal dependencies, can predict events such as arrhythmia and hypertension from physiological time-series data such as heart rate and blood pressure. Long Short-Term Memory (LSTM) networks are RNN variants that maintain long-term dependencies across time steps. LSTMs have accurately detected arrhythmias through sequential pattern recognition in ECG signals [11,24]. Deep Belief Networks (DBNs) are the foundation of the Deep Red Fox Belief Prediction System (DRFBPS), which extracts hierarchical features from complex datasets. For instance, the DRFBPS can predict heart disease based on various indicators such as age, blood pressure, and cholesterol [20].

Autoencoders are unsupervised learning models employed in dimensionality reduction and feature extraction tasks. When integrated into predictive models for healthcare analytics, autoencoders enhance the performance by isolating the most relevant features from high-dimensional datasets [17].

Ensemble methods combine multiple learning models to improve the prediction accuracy and robustness. The authors of Ref. [22], who constructed a risk prediction index of diabetes based on UK Biobank data, found that ensemble approaches outperform their individual constituent models in terms of precision and generalization on multiple test groups.

As shown in Table 4, tree-based ensemble methods (RF, XGBoost, LightGBM) dominated structured-data contexts, reflecting their robustness and scalability. However, their frequent pairing with ICU datasets raises the question of whether their success is model-driven or simply data-driven. Deep learning models (CNN, RNN, LSTM) performed best in domains requiring pattern recognition from unstructured signals (e.g., imaging, ECG), showing that data modality, rather than disease type, is the decisive factor in model selection. Notably, ensemble combinations often outperformed individual models, reinforcing the view that no single algorithm is universally optimal across healthcare domains.

Constructed from Table 1 and Table 4, the table below connects each domain with the ML model used, and the best model observed for each domain.

As shown in Table 5, most reviewed studies were concentrated in ICU and critical care, where ensemble and tree-based models such as XGBoost and RF consistently achieved strong predictive performance for sepsis and mortality but struggled with generalizability across populations [4,8,10]. Emergency Department (ED) applications applied deep learning models on triage and vital-sign data, achieving promising sensitivity in early risk stratification [12,14]. In contrast, cardiovascular diseases and oncology were underrepresented, but those studies demonstrated the added value of imaging- and genomic-based deep learning frameworks [11,21]. Chronic diseases and COVID-19 relied on IoT-enhanced ML pipelines and DL classifiers, though many lacked prospective validation [2,16]. Finally, general healthcare and other conditions (e.g., AKI, length-of-stay prediction) showed effective use of risk scoring and Gradient Boosting, but highlighted persistent challenges with EHR integration and external reproducibility [15,19].

### 3.5. Evaluation Metrics

Performance evaluation was closely tied to clinical priorities. ICU and ED studies prioritized sensitivity and F1-score, where missing a critical case would have dire consequences, whereas oncology and survival analysis leaned on C-index and Brier scores, reflecting the longitudinal nature of disease progression. While AUROC remained the most reported metric, its dominance risks obscuring clinically relevant trade-offs such as alarm fatigue from low specificity.

AutoML (AutoPrognosis 2.0) has also delivered promising results in diagnostic modeling with minimal manual tuning (see, for example, [22]). Performance metrics are not chosen arbitrarily, but must satisfy the core clinical needs in risk-sensitive environments. For instance, sensitivity and specificity are critical in ICU and emergency settings, where missing a high-risk patient (false negative) or generating unnecessary alarms (false positive) can produce serious consequences [4,5,10,12].

The high frequency of AUC reporting demonstrates the importance of capturing the overall discriminative power, especially on imbalanced datasets [1,7,14]. Rare events or deteriorations tended to be evaluated with the F1-score and precision-recall metrics [8,13,18], while survival prediction papers appropriately used time-to-event metrics such as the C-index and integrated Brier score [19,21]. This alignment shows that evaluations of healthcare ML models are not merely technical but are driven by clinical context, risk tolerance, and decision-making priorities. Most of the studies relied on common performance metrics such as AUC and F1-score [2,6,10,14,16,23], and the performance indices used in the reviewed studies are listed in Table 6.

The metrics that were most commonly reported include AUC/AUROC/ROC, F1-score, accuracy, and recall/sensitivity. Each of these metrics appeared in more than ten studies, indicating that they are often considered standard and reliable performance metrics for ML models in healthcare, especially in classification tasks such as sepsis [1,4,5,10,14]. The F1-score is particularly important as it balances precision and recall, which is crucial in healthcare scenarios where incorrect predictions can result in significant clinical risks.

Some studies employed the precision (PPV), specificity, and negative predictive value (NPV) metrics, which are often critical in diagnostic applications or when minimizing false alarms in clinical settings [9,11,21]. Fewer studies utilized advanced metrics—such as the Brier score, C-index, decision curve analysis, integrated AUC, and integrated Brier score—which are typically used in probabilistic models or time-to-event (survival) analyses [19,21,24].

Studies using multi-metric evaluations (e.g., [20]) produced the most nuanced insights, suggesting that future work should adopt domain-adaptive evaluation frameworks rather than relying on generic metrics.

## 4. Discussion

**Figure 3:**
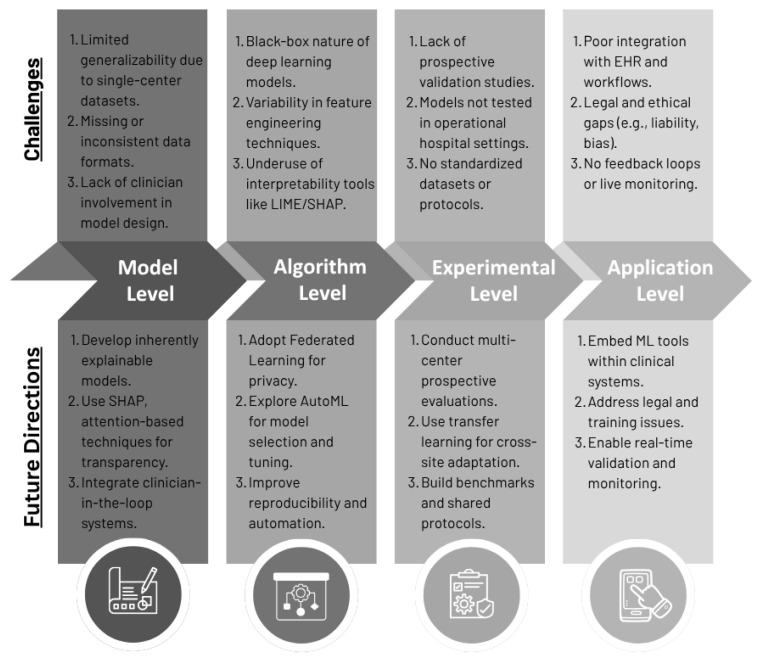
Challenges and future directions of ML in predictive health care.

In this section, we will explore the challenges associated with the implementation of ML models in healthcare and outline potential future directions for research. Figure 3 illustrates the mapping of these challenges to future research directions.

### 4.1. Challenges and Issues

To provide a comprehensive perspective for researchers and practitioners working in predictive healthcare analytics, we categorized the observed challenges into four key concerns: data and generalizability, algorithm and interpretability, clinical integration, and privacy and regulatory issues. Each of these concerns has limited the widespread clinical translation of ML-based predictive models.

#### 4.1.1. Data and Generalizability

Most of the reviewed studies stated a lack of external generalizability as a major concern. Many models were developed using retrospective, single-center datasets such as MIMIC and PhysioNet, which are consistent but lack diverse patient demographics, clinical protocols, and regional healthcare practices [5,8,17]. Models based on non-diverse datasets usually lose performance when applied to different institutions or populations. Moreover, data imbalance—especially when predicting rare events such as early-stage sepsis or ICU mortality—limits the training and evaluation of models [2,7]. Other common problems are missing values, heterogeneous data formats, and inconsistent preprocessing protocols. These problems severely degrade the robustness of models and increase the difficulty of inter-study comparisons.

#### 4.1.2. Algorithm and Interpretability

Although complex models such as deep neural networks and ensemble architectures deliver superior performance, their black-box nature remains a critical obstacle to clinical adoption. Many clinicians hesitate to use non-interpretable models, especially when the outcomes affect high-risk decisions [1,6]. Techniques such as SHapley Additive exPlanations (SHAP) and Local Interpretable Model-agnostic Explanations (LIME), which improve the interpretability of models, remain underutilized and often cannot explain the model behavior in real-time. In addition, feature engineering methods varied widely across the studies, ranging from manual statistical aggregation to autoencoders and domain-guided selection, adding another layer of variability.

#### 4.1.3. Clinical Integration and Real-World Use

Despite their strong retrospective performance, prospective validation and real-time deployment were rarely attempted in the reviewed studies. ML models are rarely tested in operational hospital systems or embedded into EHRs [3,9]. Moreover, clinician-in-the-loop frameworks were missing from most pipelines, reducing opportunities for user feedback and trust building. Models built without consulting healthcare professionals tended to select irrelevant features or produce outputs not aligned with real clinical priorities. These gaps highlight the lack of collaboration between ML developers and clinical end-users.

#### 4.1.4. Privacy, Ethics, and Regulatory Issues

Given the sensitive nature of patient data, privacy compliance under the Health Insurance Portability and Accountability Act (HIPAA) or the General Data Protection Regulation (GDPR) poses a major challenge. Federated learning has been explored as a privacy-preserving approach, but has been fully implemented only in a minority of studies. In addition, few studies have addressed the ethical considerations around automated decision-making in healthcare, including algorithmic bias, transparency of predictions, and accountability for incorrect predictions. Without proper governance, ML models might reinforce existing health disparities or introduce new risks to patient care [13,20].

### 4.2. Future Research Directions

To fully exploit the potential of ML in predictive healthcare analytics, future research directions must meet the challenges identified at the model, algorithm, experimental, and application levels. Collectively resolving these dimensions would accelerate the clinical translation of ML models and enhance their usability, safety, and equity in real-world healthcare settings.

#### 4.2.1. Model Level

Many ML models deliver strong predictive capabilities, but their limited interpretability remains a key barrier to adoption in clinical practice [14,21]. Enhancing the explainability of models through methods such as SHAP and attention-based visualization is an important future research direction [21,22]. The development of inherently interpretable models tailored to clinical decision-making is essential for fostering clinician trust and regulatory acceptance [14]. Moreover, implementing clinician-in-the-loop designs may improve the integration of model insights into real-time decision support systems [21].

#### 4.2.2. Algorithm Level

Ensuring patient data privacy while maintaining model performance is a growing concern, especially across healthcare institutions. One promising solution is federated learning, which enables decentralized training without direct data sharing [24]. Automatic ML platforms such as AutoPrognosis should be investigated for automating the model selection, hyperparameter tuning, and pipeline optimization [22], thus democratizing access to ML among non-technical stakeholders. These platforms can considerably reduce the development time and improve the reproducibility of the model [22].

#### 4.2.3. Experimental Research Level

Current studies are largely limited to retrospective or short-term analyses in isolated environments [15]. ML models should be tested in prospective, multi-institutional validation studies under real-world constraints [15,24]. Models employing transfer learning and domain adaptation techniques would maintain robust performance across diverse populations and clinical contexts [13]. Moreover, the establishment of benchmark datasets and standardized protocols is important for meaningful inter-study comparisons and enhanced reproducibility [22].

#### 4.2.4. Application Level

ML models have been insufficiently integrated into EHRs and existing clinical workflows [14,15]. Future work should focus on the seamless embedding of ML tools within hospital information systems, ensuring their usability with minimal disruption to clinical routines [11]. Overcoming the legal, ethical, and operational barriers, such as liability, user training, and alert fatigue, is also crucial for the sustained adoption of ML in clinical settings [11,15]. Finally, researchers should incorporate real-time validation and monitoring mechanisms that assess the ongoing model performance and safety in live environments [14].

## 5. Conclusions

This review aims to assess the role of machine learning in predictive healthcare, highlighting both advancements and existing challenges. Table 7 maps each research question to the sections addressed and summarizes the main findings from the reviewed studies.

The findings indicate that while ensemble methods (such as Random Forest, XGBoost, and LightGBM) and deep learning architectures demonstrate significant technical progress, their predominant application in intensive care unit (ICU) settings reveals a bias toward high-acuity, data-rich environments. This focus restricts broader clinical applications, particularly in oncology, chronic conditions, and primary care, which remain underexplored despite their importance.

Moreover, while our systematic literature review identified 22 relevant studies on predictive machine learning in healthcare, we acknowledge that this number may seem limited. Our stringent inclusion criteria were necessary to ensure the quality of the studies, but they may also introduce potential biases by excluding non-English publications and research from underrepresented regions, such as Africa, Latin America, and Eastern Europe. These geographic disparities can affect the generalizability of our findings, highlighting the need for future research to include a wider range of studies from diverse regions to create a more comprehensive understanding of predictive modeling in healthcare.

The review also identifies a trade-off between reproducibility and diversity in research. Although benchmark datasets like MIMIC and PhysioNet facilitate comparisons across studies, their overuse may perpetuate systematic biases. In contrast, local hospital datasets provide contextual validity but often lack generalizability. Therefore, hybrid validation strategies that balance these aspects are essential for developing models that are both robust and clinically applicable.

Additionally, technical complexity is not the primary barrier to progress. While interpretability frameworks (such as SHAP and LIME), AutoML platforms, and collaborative learning approaches are available, they are often underutilized. The critical challenges lie in the need for prospective validation, designs that incorporate clinician feedback, and frameworks that ensure patient safety and ethical accountability.

In conclusion, advancing predictive healthcare requires a shift from merely developing high-performing algorithms to integrating trustworthy, transparent, and generalizable systems into real-world clinical workflows. By addressing these systemic challenges and recognizing the need for broader research inclusivity, machine learning can evolve from academic research to provide equitable and scalable improvements in healthcare delivery.

## Figures and Tables

**Table 1:** Coverage of healthcare domains in the reviewed literature.

Healthcare Domain	References
ICU and Critical Care	[4,6,8,10,19]
Emergency Department (ED)	[5,12,14]
Cardiovascular Diseases	[11,20]
Oncology	[17,21]
Diabetes and Chronic Diseases	[2,22]
COVID-19	[16,17]
General Healthcare/Primary Care	[15,18,23]
Other Specific Conditions	[2,7,19]

**Table 2:** Benchmark datasets used in the reviewed studies.

Dataset	Description	References
MIMIC-III	Medical Information Mart for Intensive Care (MIMIC) is an openly available database comprising de-identified health-related data for over 40,000 patients admitted to critical care units at the Beth Israel Deaconess Medical Center between 2001 and 2012. It includes demographics, bedside vital signs (recorded approximately hourly), lab results, procedures, medications, clinical notes, imaging reports, and mortality outcomes. Available at https://mimic.physionet.org/ (accessed on 19 September 2025)	[7,24]
MIMIC-IV	An updated version of MIMIC-III, this openly available database contains ICU records from the same hospital between 2008 and 2019. It reflects a more modern healthcare practice and integrates structured clinical data with improved coding. Available at https://mimic.physionet.org/ (accessed on 19 September 2025)	[4,5,8,10,12]
PhysioNet	This dataset was developed for the PhysioNet 2019 Challenge on early prediction of sepsis. It includes time-series data from over 40,000 ICU patients across various hospital systems. Each record contains hourly vital signs (HR, MAP, Resp, O2Sat, Temp), demographics, and timestamps. The goal was to predict sepsis onset within a 6-hour window. Available at https://physionet.org/content/challenge-2019/1.0.0/ (accessed on 19 September 2025)	[9]
eICU	This dataset includes over 200,000 ICU admissions from 208 U.S. hospitals between 2014 and 2015. It is used alongside MIMIC-IV for external validation in some studies and provides rich clinical data for predictive model development. Available at https://eicu-crd.mit.edu/ (accessed on 19 September 2025)	[10]

**Table 3:** Feature extraction methods used in the reviewed studies.

Feature Extraction Method	Feature Type	References
Manual Statistical Aggregation (mean, std, etc.)	Time-series features (e.g., vitals, lab data)	[4,8,19]
Recursive Feature Elimination (RFE)	Mixed clinical features (structured data)	[2,11,13]
LASSO	High-dimensional EHR features	[10,16]
Boruta	Structured EHR data (relevant variables)	[7,16]
Principal Component Analysis (PCA)	Imaging or physiological features (dimensionality reduction)	[11]
Autoencoders	Signal-based or imaging features (latent representations)	[11,17]
SHAP-based Feature Importance	Model-agnostic feature importance across various types	[5,10,22]
Filter Methods/Information Gain	Discrete clinical and categorical features	[8]
Expert/Clinically Selected Features	Clinical scores, demographic and risk factors	[4,12,14]
Feature-Aligned Transfer Learning (FATL)	Harmonized features from heterogeneous datasets	[13]

**Table 4:** Machine learning models used in the reviewed studies.

ML Model	Description	References
Random Forest (RF)	Ensemble of decision trees combined to enhance prediction accuracy.	[4,6,8,10]
Logistic Regression (LR)	Predicts probability of binary outcomes.	[8,23]
Support Vector Machine (SVM)	Finds the optimal hyperplane separating two classes using support vectors.	[8,12]
XGBoost	Algorithm known for efficiency and high performance on large datasets.	[4,8]
Neural Networks (generic)	Deep-learning models effective for complex, high-dimensional data.	[11,24]
Decision Tree	Simple supervised models that split data by feature values using tree-like decision rules.	[4,8]
Gradient Boosting	Sequential ensemble method for classification and regression that minimizes prediction errors.	[4,8]
Naive Bayes	Probabilistic classifier effective when features are conditionally independent.	[8,19]
K-Nearest Neighbors (k-NN)	Assigns a label based on majority class of nearest neighbors	[8]
Multi-Layer Perceptron (MLP)	Feedforward neural network with input, hidden, and output layers.	[4,8]
LightGBM	Optimized gradient-boosting algorithm with leaf-wise tree growth for faster training and improved accuracy.	[4]
Convolutional Neural Networks (CNNs)	Deep learning models for processing spatial data.	[11]
Recurrent Neural Networks (RNNs)	Process sequential data to capture temporal dependencies.	[24]
Long Short-Term Memory (LSTM)	RNN variant that captures long-term dependencies over time.	[24]
Deep Belief Network (DBN)	Extract hierarchical features from complex datasets.	[20]
Autoencoder	Unsupervised models for dimensionality reduction and feature extraction.	[17]
Ensemble Methods (Custom)	Generally high accuracy and robust predictions due to combining multiple models; reduces overfitting compared to single models.	[22]

**Table 5:** Coverage of healthcare domains, ML models used, and best performing models in the reviewed studies.

Domain	ML Models	Best Model(s)	No. of Studies	References
ICU and Critical Care	RF, XGBoost, LR, Ensemble	XGBoost, Ensemble	5	[4,6,8,10,19]
Emergency Department (ED)	Deep Learning, RF	Deep Learning	3	[5,12,14]
Cardiovascular Diseases	LR, DL (imaging), RF	DL (imaging)	2	[11,20]
Oncology	ML + Genomic, Survival models	Integrated genomic ML	2	[17,21]
Diabetes and Chronic Diseases	IoT + ML pipelines, RF, SVM	RF/IoT–ML hybrid	2	[2,22]
COVID-19	RF, DL, Ensemble	DL-based classifiers	2	[16,17]
General Healthcare/ Primary Care	Risk scoring models, RF, LR	Risk scoring + RF	3	[15,18,23]
Other Specific Conditions	GBM, RF	GBM	3	[2,7,19]

**Table 6:** Evaluation metrics used in the reviewed studies.

Evaluation Metric	Description	No. of Studies	References
AUC/AUROC/ ROC	It evaluates a model’s ability to differentiate between positive and negative cases, also with imbalanced data.	15	[2,3,4,5,6,8,9,10,11,14,16,17,19,20,23]
F1-score	It balances precision and recall, making it ideal for scenarios where both false positives and false negatives can have serious clinical consequences.	13	[2,3,4,5,6,8,9,10,11,14,17,20,23]
Accuracy	It is commonly reported but can be misleading in healthcare with imbalanced data; it may appear high even when critical positive cases are missed.	12	[2,3,4,5,9,14,16,17,19,20,21,23]
Recall/Sensitivity	It measures the model’s ability to correctly identify true positive cases, which helps ensure that high-risk patients are not missed.	11	[2,3,4,6,8,9,11,14,20,21,23]
Precision/PPV	It measures how many of the predicted positive cases are actually true positives, helping to reduce false alarms and avoid unnecessary treatments.	10	[2,3,9,10,11,14,16,19,20,23]
Specificity	It measures the model’s ability to correctly identify negative cases, helping to prevent unnecessary worry for patients who do not have the condition.	4	[6,14,21,22]
Negative Predictive Value	NPV represents the proportion of negative predictions that are correct.	3	[6,14,22]
Brier Score	It measures the accuracy of predicted probabilities.	3	[14,22,24]
C-index	It is suitable for time-to-event or survival prediction models.	3	[21,22,24]
AUPRC	It focuses on the precision-recall tradeoff, especially useful for imbalanced datasets.	3	[5,8,21]
Calibration Curves	It assess whether predicted probabilities match observed outcomes.	2	[14,24]
Error Rate	It is the overall proportion of incorrect predictions.	1	[20]
Confidence Interval	CI indicates the uncertainty range around metric estimates.	1	[20]
*p*-value	It is used for hypothesis testing to determine statistical significance.	1	[20]
Decision Curve Analysis	DCA evaluates the clinical benefit of predictive models across different threshold probabilities.	1	[14]
Integrated Brier Score	IBS is an integrated measure of prediction accuracy over time.	1	[22]
Integrated AUC	iAUC is the time-integrated area under the ROC curve, assessing model performance longitudinally.	1	[22]
Absolute Calibration Error	ACE measures the absolute difference between predicted probabilities and observed outcomes.	1	[8]

**Table 7:** Mapping of included studies to the research questions (RQs).

RQ	Addressed In	Summary of Findings
RQ1	Healthcare Domains (Section 3.1)	Predictive ML has been applied in various healthcare domains such as ICU (sepsis, mortality), oncology, cardiology, diabetes management, and COVID-19. ICU and chronic disease studies [4,6,8,10,19] were the most frequent focus.
RQ2	Machine Learning Models (Section 3.4)	The most commonly used algorithms were Random Forest (RF) [4,6,8,10]. Deep learning models such as CNNs and LSTMs were increasingly applied in imaging and time-series tasks.
RQ3	Evaluation Metrics (Section 3.5)	More than half of the studies used metrics such as AUC, accuracy, and F1-score [2,3,4,5,6,8,9,10,11,14,16,17,19,20,23]. Some studies also reported calibration metrics such as the C-index and Brier score [22,24], particularly in survival analysis.
RQ4	Challenges and Issues (Section 4.1)	Key challenges included limited and imbalanced datasets, lack of generalizability across populations, poor interpretability of models, integration difficulties into clinical practice, and privacy concerns.
RQ5	Future Research Directions (Section 4.2)	Future directions highlighted the need for federated learning to address privacy, AutoML for easier adoption, and integration with clinical workflows for real-world deployment.

## Data Availability

No new data were created or analyzed in this study.
